# Association of systemic inflammation index with survival in patients with advanced perihilar cholangiocarcinoma treated with interventional therapy

**DOI:** 10.3389/fonc.2022.1038759

**Published:** 2022-12-22

**Authors:** Jinyu Li, Long Gao, Tianci Liu, Duiping Feng

**Affiliations:** ^1^ Department of Oncological and Vascular Intervention, First Hospital of Shanxi Medical University, Taiyuan, China; ^2^ College of Medical Imaging, Shanxi Medical University, Taiyuan, China

**Keywords:** advanced perihilar cholangiocarcinoma, systemic immune-inflammation index, overall survival, prognosis, interventional therapy

## Abstract

**Objective:**

Immunity and inflammation are key mediators of carcinoma development, invasion and metastasis. However, it remains unknown whether the systemic immune-inflammation index (SII) can be used as a prognostic indicator for cholangiocarcinoma. In this study, we investigated the association and predictive value of the SII with the prognosis of advanced perihilar cholangiocarcinoma (pCCA) after interventional therapy.

**Methods:**

A retrospective cohort of patients with advanced pCCA treated with interventional therapy at the First Hospital of Shanxi Medical University enrolled in this study from January 2019 through January 2021 was examined. Cox regression models were used to analyze the relationship between the SII and overall survival (OS) of patients with advanced pCCA. Receiver operating characteristic (ROC) analysis was used to evaluate the predictive power of SII.

**Results:**

Preoperative SII was positively associated with poor OS of pCCA after interventional therapy, with corresponding hazard ratios (HR) of 1.57 (95% CI: 1.17 - 2.10) for an inter-quartile range increase. The predictive power of SII was higher than that of other inflammation indexes based on ROC analysis (AUC = 0.835 [95% CI (0.731 - 0.940)]). The optimal cut-off values, sensitivity, and specificity with SII were 700, 0.774 and 0.846, respectively. An SII ≥ 700 was significantly associated with lymph node metastasis and high carbohydrate antigen199 (CA199) level. In multivariate analyses, total bilirubin, carbohydrate antigen 199, vascular invasion, and SII independently predicted overall survival (P < 0.05).

**Conclusion:**

This is the first study demonstrating that an increase in the SII is associated with poor advanced pCCA prognosis, and could serve as a reliable prognostic indicator of pCCA after interventional therapy.

## Introduction

Cholangiocarcinoma (CCA) is a rare digestive malignancy with limited therapeutic options and a poor prognosis (1). It is anatomically classified as intrahepatic, perihilar, and distal. Of these, perihilar cholangiocarcinoma (pCCA) accounts for 50-60% of all cholangiocarcinoma (2). Approximately 90% of pCCA patients present with biliary symptoms, including, most commonly, painless jaundice (3). Jaundice often reflects locally advanced or metastatic disease. Since early symptoms are not obvious, most patients have advanced-stage disease at presentation. Only 35% patients have early stage disease that is amenable to surgical resection with curative intent (4). For advanced pCCA, interventional therapy (percutaneous transhepatic cholangial drainage (PTCD) combined with hepatic artery infusion chemotherapy (HAIC)) can reduce jaundice and control the tumor size, which has become an important locoregional treatment and palliative care (5–7). It is important to identify patient subpopulations with poor prognosis for interventional therapy to optimize adjuvant treatments and provide new treatments to these subpopulations without delay.

The reasons for the poor prognosis in pCCA after interventional therapy are complex and multifactorial. It is largely recognized that poor prognosis in CCA are associated with immune and inflammation states (8, 9). Immune and inflammatory cells such as neutrophils, platelets, and lymphocytes also contribute to tumor cell invasion into the peripheral blood, where the tumor cells can survive and reseed distant organs. The systemic immune-inflammation index (SII), based on lymphocyte, neutrophil, and platelet counts, reflected comprehensively the balance of host inflammatory and immune status, which was developed to identify patients with poor prognosis (10, 11). Several studies have shown that SII is closely related to the prognosis of tumor and have some prognostic value in a variety of malignancies, including hepatocellular carcinoma (10), colon cancer (12), cervical cancer (13) and distal cholangiocarcinoma (14). However, for patients with advanced pCCA treated with interventional therapy, there is no data related to the relationship between prognosis and SII.

In this study, we investigated the relationship of between SII and the prognosis of advanced pCCA after interventional therapy. And the potential prognostic value of SII in patients with advanced pCCA who underwent interventional therapy was also evaluated in this study.

## Patients and methods

### Study population

This is a single-center retrospective study. The advanced pCCA refers to patients who cannot be resected with a negative margin (R0) and have no distant metastasis (locally advanced). In total, 76 patients with advanced pCCA who had undergone interventional therapy in the Department of Oncological and Vascular Intervention of the First Hospital of Shanxi Medical University from January 2019 to January 2021 were reviewed, of whom 10 were excluded due to uncompleted clinical information. Thus, the present retrospective study comprised 66 patients with advanced pCCA. Demographic and clinicopathological characteristics (age, gender, liver function, tumor markers, inflammation-based prognostic scores, tumor size, vascular invasion, and lymph node invasion) were collected from the medical records, and the study was approved by the Research Ethics Committee of the First Hospital of Shanxi Medical University. The inclusion criteria were that patients were ≥18 years of age, had received a histopathologic diagnosis of pCCA, were unable to a negative margin (R0) resection, and were treated with PTCD combined with HAIC. Exclusion criteria were: (1) patients with distant metastasis and recurrent pCCA after resection, (2) patients undergoing palliative resection, chemotherapy, radiotherapy and other anti-tumor treatments before this study, (3) patients with other inflammatory related diseases, blood diseases, immune diseases, and other malignant tumors, (4) patients with biliary stent and radioactive particle implantation, (5) using drugs that affect peripheral blood cell in recent 1 months, (6) patients with missing data, difficult follow-up and unknown cause of death.

### Procedure and treatment

The PTCD and HAIC procedure was carried out according to the method described by Takada T et al. (15) and Wang X et al. (7). All patients underwent PTCD (routine placement of external drainage tube) and HAIC (local perfusion chemotherapy with 30 mg of lobaplatin and 1 g of gemcitabine pumped into the intrinsic hepatic artery; 1 cycle every 3-4 weeks, up to 6 cycles). After PTCD, sequential HAIC treatment was carried out according to the doctor’s judgment.

### Definition of inflammation-based prognostic scores

The neutrophil-lymphocyte ratio (NLR) values were the ratio of neutrophil count and lymphocyte count. The platelet-lymphocyte ratio (PLR) values were the ratio of platelet count and lymphocyte count. The monocyte-lymphocyte ratio (MLR) values were the ratio of monocyte count and lymphocyte count. The SII was calculated as platelet * neutrophil/lymphocyte. The systemic inflammatory response index (SIRI) was calculated as (neutrophil * monocyte)/lymphocyte.

### Statistical analysis

All analyses were conducted using SPSS (version 24.0) and SAS software (version 9.4). Before analysis, normality tests or P-P plots were used to check the normality of the variables. Continuous variables are expressed as mean ± standard deviation (SD) or median (inter-quartile range). Categorical variables are described as frequency (proportion). Cox proportional hazards models were used to analyze the relationship between the inflammation-based prognostic scores and overall survival (OS) of patients with advanced pCCA, and restricted cubic spline models were further used to examine the shapes of the dose-response association. Survival curves were plotted by Kaplan-Meier method and compared by Log-Rank test to compare between-group variability. Receiver operating characteristic (ROC) analysis was used to evaluate the predictive power of SII. In all cases, bilateral P values less than 0.05 were considered statistically significant.

## Results

### Characteristics of the participants in this study

Of the 66 participants, 36 (54.5%) were male. The ages ranged from 35 to 84 years, with a mean of 64.0 years. The detailed baseline demographic and clinicopathological characteristics of the study population are listed in **Table 1** and **Supplementary Table S1**.

**Table 1:** Demographics and clinicopathological characteristics in this study (n=66).

Variables	Values
Age (years)^a^	64.0 ± 1.2
Sex (Male)	36 (54.5%)
ALT (U/L)^b^	110.5 (51.0, 134.0)
AST (U/L)^b^	120.0 (64.5, 159.3)
ALB (g/L)^b^	32.6 (29.1, 34.9)
TBIL (μmol/L)^b^	276.8 (185.5, 342.8)
CEA (μg/L)^b^	3.9 (1.9, 6.2)
CA199 (U/ml)^b^	305.1 (105.1, 1616.4)
CA125 (U/ml)^b^	33.5 (13.7, 80.6)
Tumor size (cm)^b^	3.2 (2.3, 4.9)
Lymph node metastasis (yes)	37 (56.1%)
Vascular invasion (yes)	13 (19.7%)
Overall survival (days)^b^	224.5 (163.0, 290.3)
NLR^b^	4.2 (2.0, 7.1)
PLR^b^	190.4 (132.2, 270.0)
MLR^b^	0.5 (0.3, 0.8)
SII^b^	1049.2 (430.8, 1685.3)
SIRI^b^	2.8 (1.0, 4.5)

^a^ mean ± SD; ^b^ Median with inter-quartile range.

AST, aspartate aminotransferase; ALT, alanine aminotransferase; TBIL, total bilirubin; ALB, albumin; CEA, carcinoembryonic antigen; CA199, carbohydrate antigen199; CA125, carbohydrate antigen 125; NLR, neutrophil-lymphocyte ratio; PLR, platelet-lymphocyte ratio; MLR, monocyte-lymphocyte ratio; SII, systemic immune-inflammation index; SIRI, systemic inflammatory response index. Tumor size refers to the maximum diameter of the tumor; Lymph node metastasis and vascular invasion are evaluated by imaging.

### The association of between inflammation-based prognostic scores and prognosis of pCCA after interventional therapy

**Figure 1:**
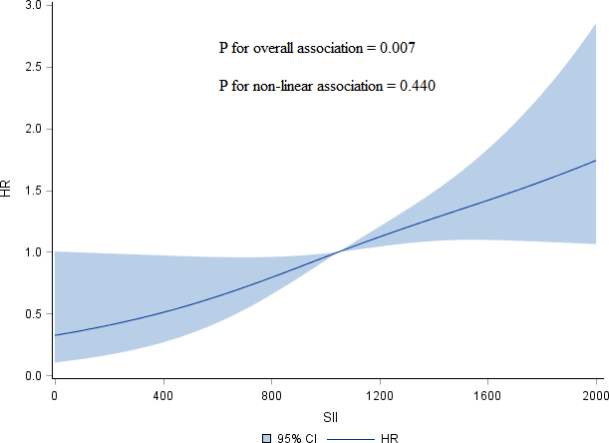
Restricted cubic spline models of association of SII with HR of overall survival for advanced pCCA after interventional therapy adjusted for age, sex, TBIL, CA199, lymph node metastasis, and vascular invasion. SII, systemic immune-inflammation index; HR, hazard ratio; pCCA, perihilar cholangiocarcinoma; TBIL, total bilirubin; CA199, carbohydrate antigen199.

According to the distribution of inflammation-based prognostic scores, we classified participants into 4 groups: Q1 (the 1st inter-quartile range (IQR)), Q2 (the 2nd IQR), Q3 (3rd IQR), and Q4 (the 4th IQR). The different models used to test the association between inflammation-based prognostic scores and HR of overall survival in advanced pCCA is presented in **Table 2**. We consistently observed that a monotonic and positive association of SII with HR of overall survival in different models, and p-value for trend test was less than 0.05. Further, the dose-response association of SII with increased HR of overall survival from advanced pCCA was confirmed in the restricted cubic spline models after adjusting for potential confounders (**Figure 1**). However, there were no significant trends among the HR of overall survival in participants stratified by NLR, PLR, MLR, and SIRI. These data suggested that preoperative SII was associated with the prognosis of advanced pCCA after interventional therapy and may be used as an early predictor to predict the prognosis and survival of advanced pCCA.

**Table 2:** Associations between inflammation-based prognostic scores and the prognosis of pCCA after interventional therapy by cox regression in different models.

		Hazard ratio (95% CI)		
		Model 1	Model 2	Model 3
NLR	Q1	1 (Ref.)	1 (Ref.)	1 (Ref.)
	Q2	1.52 (0.63 - 3.67)	1.52 (0.63 - 3.70)	1.22 (0.49 - 3.04)
	Q3	3.80 (1.59 - 9.10)	3.77 (1.56 - 9.13)	1.71 (0.64 - 4.56)
	Q4	2.51 (1.07 - 5.89)	2.42 (1.0 - 5.90)	1.74 (0.69 - 4.41)
	P for trend	**0.010**	**0.015**	0.198
PLR	Q1	1 (Ref.)	1 (Ref.)	1 (Ref.)
	Q2	1.31 (0.57 - 3.04)	1.31 (0.55 - 3.12)	1.17 (0.45 - 3.04)
	Q3	1.80 (0.81 - 4.02)	1.80 (0.80 - 4.09)	1.24 (0.51 - 2.98)
	Q4	2.66 (1.19 - 5.96)	2.65 (1.08 - 6.52)	1.80 (0.73 - 4.47)
	P for trend	**0.010**	**0.022**	0.203
MLR	Q1	1 (Ref.)	1 (Ref.)	1 (Ref.)
	Q2	1.31 (0.58 - 2.92)	1.29 (0.58 - 2.91)	0.95 (0.41 - 2.20)
	Q3	2.34 (1.06 - 5.21)	2.34 (0.96 - 5.70)	1.21 (0.46 - 3.16)
	Q4	2.60 (1.16 - 5.80)	2.65 (1.18 - 5.94)	1.74 (0.73 - 4.14)
	P for trend	**0.009**	**0.011**	0.127
SII	Q1	1 (Ref.)	1 (Ref.)	1 (Ref.)
	Q2	1.25 (0.52 - 3.01)	1.15 (0.46 - 2.85)	0.99 (0.40 - 2.47)
	Q3	3.15 (1.33 - 7.47)	3.32 (1.40 - 7.92)	1.50 (0.56 - 4.04)
	Q4	4.04 (1.69 - 9.65)	4.76 (1.87 - 12.13)	3.24 (1.22 - 8.62)
	P for trend	**<0.001**	**<0.001**	**0.008**
SIRI	Q1	1 (Ref.)	1 (Ref.)	1 (Ref.)
	Q2	1.44 (0.64 - 3.26)	1.44 (0.64 - 3.25)	1.37 (0.59 - 3.17)
	Q3	2.45 (1.07 - 5.62)	2.31 (0.90 - 5.91)	2.21 (0.83 - 5.90)
	Q4	3.26 (1.44 - 7.37)	3.44 (1.50 - 7.88)	1.88 (0.77 - 4.57)
	P for trend	**0.002**	**0.002**	0.140

Model 1: unadjusted for covariates. Model 2: adjusted for age and sex. Model 3: adjusted for age, sex, total bilirubin, carbohydrate antigen199, lymph node metastasis, and vascular invasion. The bold presented a p-value less than 0.05. NLR, neutrophil-lymphocyte ratio; PLR, platelet-lymphocyte ratio; MLR, monocyte-lymphocyte ratio; SII, systemic immune-inflammation index; SIRI, systemic inflammatory response index.

### Ability of SII to predict prognosis of advanced pCCA after interventional therapy

**Figure 2:**
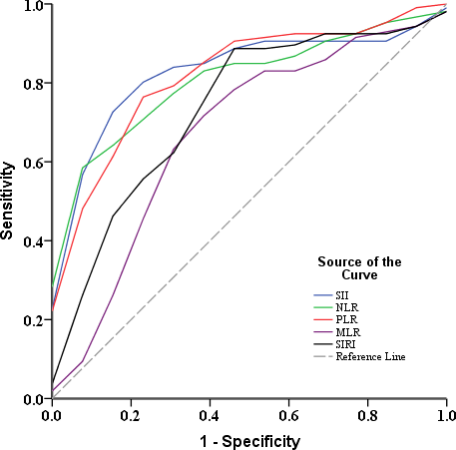
ROC curves of the probability of inflammation-based prognostic scores in predicting prognosis of advanced pCCA after interventional therapy. ROC, receiver operating characteristic; pCCA, perihilar cholangiocarcinoma; NLR, neutrophil-lymphocyte ratio; SII, systemic immune-inflammation index; PLR, platelet-lymphocyte ratio, MLR, monocyte-lymphocyte ratio; SIRI, systemic inflammatory response index.

**Figure 3:**
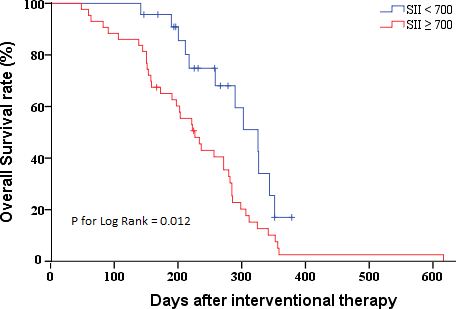
Kaplan-Meier survival curves of patients with pCCA after interventional therapy by SII distribution. pCCA, perihilar cholangiocarcinoma; SII, systemic immune-inflammation index.

After confirming the relationship between SII and pCCA prognosis after interventional therapy, we further evaluated its ability to predict pCCA prognosis. We plotted the ROC curves and found the AUC value of SII (AUC = 0.835 [95% CI (0.731 - 0.940)]) was greater than the NLR (AUC = 0.816 [95% CI (0.712 - 0.921)]), PLR (AUC = 0.828 [95% CI (0.715 - 0.941)]), MLR (AUC = 0.673 [95% CI (0.499 - 0.848)]), and SIRI (AUC = 0.735 [95% CI (0.578 - 0.893)]) (**Figure 2**). The optimal cut-off values (SII = 700) for the SII in terms of prognosis of advanced pCCA was determined, with sensitivity values of 0.774 and specificity values of 0.846. The Kaplan-Meier analysis indicated that the high SII (SII ≥ 700) was associated with shorter overall survival (P for log-rank = 0.012) (**Figure 3**). The median of overall survival was significantly higher in patients in the SII < 700 group than those in the SII ≥700 group (324 days *vs.* 225 days).

### The correlation between the SII and clinicopathological characteristics

**Figure 4:**
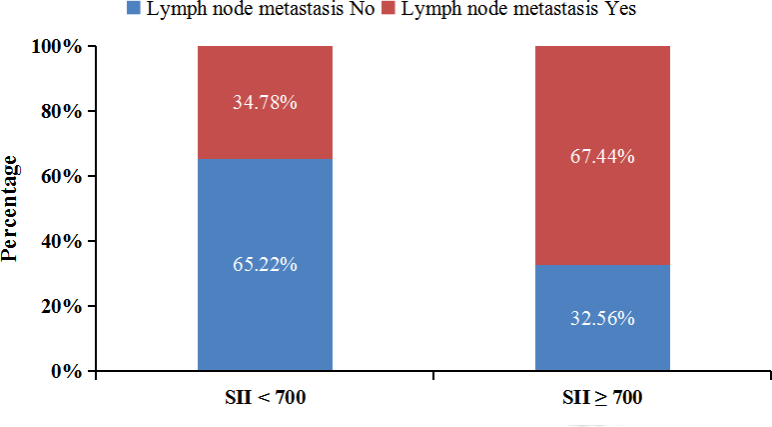
Lymph node metastasis rate of patients in different SII groups. SII, systemic immune-inflammation index.

To further clarify the reasons for the association of SII with the prognosis of advanced pCCA, we investigate the association of SII with the clinicopathological characteristics of advanced pCCA. We found that SII was associated with lymph node metastasis and the carbohydrate antigen199 (CA199) level. Compared with SII < 700 group, participants in the SII ≥ 700 group had higher CA199 level (P = 0.010) (**Table 3**). Scatter plot analyses also revealed a significant positive correlation between the SII and CA199 level (r = 0.306; P = 0.001, **Supplementary Figure S1**). And the SII ≥ 700 group had more participants with lymph node metastasis than those in SII < 700 group (67.44% *vs.* 34.78%) (**Figure 4**).

**Table 3:** Clinicopathological characteristics of participant in different SII group.

Variables^a^	SII		P
	<700	≥ 700	
Age (years)	66.0 (61.0, 74.0)	64.0 (57.0, 69.0)	0.066
ALT (U/L)	78.0 (51.0, 150.0)	112.0 (50.0, 129.0)	0.952
AST (U/L)	89.0 (62.0, 154.0)	132.0 (68.0, 186.0)	0.269
ALB (g/L)	33.8 (30.2, 39.6)	32.2 (28.4, 33.9)	0.068
TBIL (μmol/L)	268.7 (122.0, 355.4)	276.8 (251.2, 338.6)	0.419
CEA (μg/L)	3.2 (2.3, 4.8)	4.5 (1.7, 7.2)	0.244
CA199 (U/ml)	221.0 (52.0, 353.7)	578.0 (123.0, 3745.0)	**0.010**
CA125 (U/ml)	24.2 (13.7, 80.6)	43.7 (12.0, 80.6)	0.364
Tumor size (cm)	2.7 (1.9, 4.8)	3.3 (2.5, 5.1)	0.155

^a^Median with inter-quartile range. The bold presented a p-value less than 0.05.

AST, aspartate aminotransferase; ALT, alanine aminotransferase; TBIL, total bilirubin; ALB, albumin; CEA, carcinoembryonic antigen; CA199, carbohydrate antigen199; CA125, carbohydrate antigen 125; SII, systemic immune-inflammation index.

### Influencing factors of prognosis after interventional therapy for advanced pCCA

On the basis of the multiple cox regression analysis by forward method of factors, total bilirubin (TBIL), CA199, vascular invasion and SII were included in the final multivariate cox regression model. A higher TBIL, CA199, and SII and vascular invasion were found to be independent predictors of advanced pCCA after the interventional therapy. Under the premise that other variables remain unchanged, each an IQR increasing in SII, the hazard ratio of overall survival for advanced pCCA increased by 1.57 times (HR = 1.57, P < 0.05) (**Table 4**).

**Table 4:** Cox regression analyses the risk factors of prognosis for advanced pCCA after interventional therapy.

Variables	b	HR (95% CI)	P value
TBIL ^a^	0.346	1.41 (1.05, 1.90)	0.022
CA199 ^a^	0.545	1.724 (1.30, 2.29)	<0.001
Vascular invasion (no vs. yes)	0.765	2.15 (1.01, 4.56)	0.046
SII ^a^	0.451	1.57 (1.17, 2.10)	0.003

^a^ HR for each additional 1 inter-quartile range. SII, systemic immune-inflammation index; TBIL, total bilirubin; CA199, carbohydrate antigen199.

## Discussion

To the best of our knowledge, this is the first report to demonstrate the prognostic value of the SII (a well-known immune inflammation index) for patients with advanced pCCA after interventional therapy. Advanced pCCA patients with a high SII before interventional therapy had a poor overall survival. Compared with inflammation-based prognostic scores (NLR, PLR, MLR, and SIRI), the SII was a powerful prognostic indicator of poor overall survival in patients with advanced pCCA after interventional therapy and was a promising tool for advanced pCCA treatment strategy decisions.

The SII was first proposed in 2014 by Hu et al. (10) as a prognostic indicator among patients with hepatocellular carcinoma. Subsequently, the role of SII in the prognosis of gastrointestinal malignancies has been repeatedly demonstrated, including intrahepatic and extrahepatic cholangiocarcinoma (16–18). But, pCCA patients are relatively scarce and mostly only included patients undergoing surgical resection. There were no studies on the association of SII and survival outcomes in patients with advanced pCCA after interventional therapy. Our conclusions were consistent with previous studies that high SII was independent predictors of poor prognosis for patients with CCA (17). But, compared with previous studies, the cut-off values of SII was different in predicting the prognosis of CCA. We believe the mainly reason was characteristics of the study population, in particular the type and severity of the disease. The cut-off values of SII reported by Hui Li et al. (19) and Jian Li et al. (20) were 450 and 412.6, respectively, which were lower than our report (SII= 700). We speculated that the cut-off value for SII was related to the stage of the disease, and the baseline level of SII was higher in our study.

Besides, the predictive power of SII was also superior to that of NLR, PLR, MLR, and SIRI. To clarify why SII can be used as a predictor, we further investigated the association of SII with demographic and clinicopathological characteristics. Notably, SII was associated with tumor-related features, such as CA199 level and lymph node metastasis. We speculate that this is related to an increase in platelets, a component of SII. Previous studies reported elevated platelet counts are significantly associated with a higher rate of lymph node metastasis, even if the platelet counts are within the reference range (21, 22). This is related to the fact that platelets can promote tumor cell migration and invasion into the surrounding microenvironment, as well as promote tumor endothelial cell arrest, extravasation and seeding. Platelets are known to contain important angiogenic factor, such as vascular endothelial growth factor (VEGF). The VEGF facilitate tumor metastasis *via* modulating the lymphatic vessels (23), and also can induce sentinel node lymphatic angiogenesis and promote lymphatic metastasis even before the tumor has metastasized (24, 25). This suggests that SII can be used as a prognostic predictor in advanced pCCA because of its association with inflammation and clinicopathological characteristics.

The mechanisms by which SII predicts survival outcomes in cancer patients are not clear. The predictive value of the SII for tumor prognosis might be elucidates by the function of the lymphocyte, neutrophil, and platelets cells, and their close relationship with tumor biomarkers (10). It is well known that inflammation plays a key role in tumorigenesis and progression, and the tumor microenvironment composed of inflammatory cells is increasingly recognized as an essential player in the tumor process. Studies shown neutrophils can promote ICC progression by activating STAT3 (26). Neutrophils can also release cytotoxic substances that cause DNA damage in epithelial cells and activate oncogenes (27), and it can suppress adaptive immune responses and reduce the level of activated lymphocytes (28). Platelets and their receptors can mediate cancer cell growth and angiogenesis. The interaction of tumor cells with platelets is a prerequisite for successful hematogenous metastatic dissemination (29). The lymphocyte is a key mediator of antitumor immunity (30). The B lymphocytes are necessary for establishing chronic inflammation, and it may regulate cancer progression by altering the levels of circulating cytokines and chemokines. The T lymphocytes not only destroy tumor cells directly, but also induce apoptosis of target cells by binding to Fas-FasL on the surface of tumor cells (31). High SII may represent an increased platelets-mediated tumor metastasis, an increased neutrophil-mediated inflammatory response, a decreased in lymphocyte-mediated antitumor immune response, and SII is a practical and comprehensive biomarker reflecting systemic inflammatory response, immune response, and tumor metastasis in tumor patients. The specific role and pathway of inflammatory response in tumor are still inconclusive, which needs further research and discussion. In addition to SII reflecting immune-inflammatory levels in patients, we also found that SII was positively associated several clinicopathological characteristics, including lymph node metastasis and high CA199 levels. CA199 is a tumor marker used in the management of gastrointestinal cancer, and elevated CA199 levels also occur in diseases of the bile ducts, especially, obstruction of the bile ducts (32). This also reflects why the predictive power of SII is better than that of NLR, because NLR only reflects the immune-inflammatory situation and does not take into account platelet-mediated tumor metastasis.

There are several limitations in this study. First, there was a rather small size sample study. But, this study gives some clues, suggesting that the prognosis predictors of interventional therapy for advanced pCCA. We should paid attention to the value of these convenient and accessible indictors in predictive pCCA prognosis. Second, this study was a single-center retrospective study. A prospective multi-center study may be required to validate our results. Third, we can not evaluate differences in therapeutic response such as RECIST between SII groups, due to missing imaging data. Finally, we did not perform a validation study on a separate patient cohort to verify the results of this study to confirm the value of the SII in pCCA prognosis. But, we conducted sensitivity analysis to validate the relationship between SII and pCCA prognosis. We obtained a consistent result in different models that pCCA patients with high SII level had poor prognosis after interventional therapy.

## Conclusion

We first found high SII was an independent predictor of a short overall survival in patients with advanced pCCA after interventional therapy. The predictive power of SII was superior to that of NLR, PLR, MLR, and SIRI. SII can be used as a prognostic predictor in advanced pCCA due to its association with inflammation and clinicopathological characteristics.

## Data availability statement

The raw data supporting the conclusions of this article will be made available by the authors, without undue reservation.

## Ethics statement

The studies involving human participants were reviewed and approved by Research Ethics Committee of the First Hospital of Shanxi Medical University. The ethics committee waived the requirement of written informed consent for participation.

## Author contributions

JL designed the study, helped to draft the manuscript, and performed the statistical analysis. LG conducted data collection, management and revision guidance. TL performed the data collection and management. DF performed research design and analytical guidance. All authors contributed to the article and approved the submitted version.

## Supplementary material

Supplementary Figure 1 Scatter plots between the SII and CA199 level. SII, systemic immune-inflammation index; CA199, carbohydrate antigen199.

## References

[1] Razumilava N Gores GJ. Cholangiocarcinoma. Lancet (London England) (2014) 21;383(9935):2168–79. doi: 10.1016/s0140-6736(13)61903-0 PMC4069226 24581682

[2] Rizvi S Khan SA Hallemeier CL Kelley RK Gores GJ. Cholangiocarcinoma - evolving concepts and therapeutic strategies. Nat Rev Clin Oncol (2018) 15(2):95–111. doi: 10.1038/nrclinonc.2017.157 28994423 PMC5819599

[3] Mansour JC Aloia TA Crane CH Heimbach JK Nagino M Vauthey JN. Hilar cholangiocarcinoma: expert consensus statement. HPB Off J Int Hepato Pancreato Biliary Assoc (2015) 17(8):691–9. doi: 10.1111/hpb.12450 PMC4527854 26172136

[4] Jarnagin WR Fong Y DeMatteo RP Gonen M Burke EC Bodniewicz BJ. Staging, resectability, and outcome in 225 patients with hilar cholangiocarcinoma. Ann Surg (2001) 234(4):507–17; discussion 517-9. doi: 10.1097/00000658-200110000-00010 11573044 PMC1422074

[5] Qi S Yan H. Effect of percutaneous transhepatic cholangial drainag + radiofrequency ablation combined with biliary stent implantation on the liver function of patients with cholangiocarcinoma complicated with malignant obstructive jaundice. Am J Trans Res (2021) 13(3):1817–24. PMC8014363 33841706

[6] Cercek A Boerner T Tan BR Chou JF Gönen M Boucher TM. Assessment of hepatic arterial infusion of floxuridine in combination with systemic gemcitabine and oxaliplatin in patients with unresectable intrahepatic cholangiocarcinoma: A phase 2 clinical trial. JAMA Oncol (2020) 6(1):60–7. doi: 10.1001/jamaoncol.2019.3718 PMC6824231 31670750

[7] Wang X Hu J Cao G Zhu X Cui Y Ji X. Phase II study of hepatic arterial infusion chemotherapy with oxaliplatin and 5-fluorouracil for advanced perihilar cholangiocarcinoma. Radiology (2017) 283(2):580–9. doi: 10.1148/radiol.2016160572 27820684

[8] Roy S Glaser S Chakraborty S. Inflammation and progression of cholangiocarcinoma: Role of angiogenic and lymphangiogenic mechanisms. Front Med (2019) 6:293. doi: 10.3389/fmed.2019.00293 PMC6930194 31921870

[9] Tamma R Annese T Ruggieri S Brunetti O Longo V Cascardi E. Inflammatory cells infiltrate and angiogenesis in locally advanced and metastatic cholangiocarcinoma. Eur J Clin Invest (2019) 49(5):e13087. doi: 10.1111/eci.13087 30767196

[10] Hu B Yang XR Xu Y Sun YF Sun C Guo W. Systemic immune-inflammation index predicts prognosis of patients after curative resection for hepatocellular carcinoma. Clin Cancer Res (2014) 20(23):6212–22. doi: 10.1158/1078-0432.Ccr-14-0442 25271081

[11] Dey S Kashav R Kohli JK Magoon R ItiShri Walian A. Systemic immune-inflammation index predicts poor outcome after elective off-pump CABG: A retrospective, single-center study. J cardiothoracic Vasc anesthesia. (2021) 35(8):2397–404. doi: 10.1053/j.jvca.2020.09.092 33046365

[12] Chen JH Zhai ET Yuan YJ Wu KM Xu JB Peng JJ. Systemic immune-inflammation index for predicting prognosis of colorectal cancer. World J gastroenterology. (2017) 23(34):6261–72. doi: 10.3748/wjg.v23.i34.6261 PMC5603492 28974892

[13] Huang H Liu Q Zhu L Zhang Y Lu X Wu Y. Prognostic value of preoperative systemic immune-inflammation index in patients with cervical cancer. Sci Rep (2019) 9(1):3284. doi: 10.1038/s41598-019-39150-0 30824727 PMC6397230

[14] Terasaki F Sugiura T Okamura Y Ito T Yamamoto Y Ashida R. Systemic immune-inflammation index as a prognostic marker for distal cholangiocarcinoma. Surg Today (2021) 51(10):1602–9. doi: 10.1007/s00595-021-02312-7 34142236

[15] Takada T Yasuda H Hanyu F. Technique and management of percutaneous transhepatic cholangial drainage for treating an obstructive jaundice. Hepato-gastroenterology (1995) 42(4):317–22. 8586361

[16] Hakeem AR Marangoni G Chapman SJ Young RS Nair A Hidalgo EL. Does the extent of lymphadenectomy, number of lymph nodes, positive lymph node ratio and neutrophil-lymphocyte ratio impact surgical outcome of perihilar cholangiocarcinoma? Eur J Gastroenterol Hepatol (2014) 26(9):1047–54. doi: 10.1097/meg.0000000000000162 25051217

[17] Liu XC Jiang YP Sun XG Zhao JJ Zhang LY Jing X. Prognostic significance of the systemic immune-inflammation index in patients with cholangiocarcinoma: A meta-analysis. Front Oncol (2022) 12:938549. doi: 10.3389/fonc.2022.938549 35875153 PMC9300870

[18] Tsilimigras DI Moris D Mehta R Paredes AZ Sahara K Guglielmi A. The systemic immune-inflammation index predicts prognosis in intrahepatic cholangiocarcinoma: an international multi-institutional analysis. HPB Off J Int Hepato Pancreato Biliary Assoc (2020) 22(12):1667–74. doi: 10.1016/j.hpb.2020.03.011 32265108

[19] Li H Wang JJ Zhang M Ren B Li JX Xu L. Prognostic significance of systemic immune-inflammation index in patients with intrahepatic cholangiocarcinoma undergoing hepatic resection. World J gastrointestinal Oncol (2020) 12(4):467–82. doi: 10.4251/wjgo.v12.i4.467 PMC7191327 32368324

[20] Li J Meng S Bo K Zhu R Wang W Liang R. Relationship between systemic immune inflammation index and postoperative prognosis of patients with hilar cholangiocarcinoma. Chin J Hepatobiliary Surg (2021) 27(2):106–9. doi: 10.3760/cma.j.cn113884-20200423-00226

[21] Qu CH Li T Tang ZP Zhu XR Han JY Tian H. Platelet count is associated with the rate of lymph node metastasis in lung adenocarcinoma. Cancer Manage Res (2020) 12:9765–74. doi: 10.2147/cmar.S273328 PMC7548228 33116836

[22] Catal O Ozer B Sit M. Prediction of lymph node metastasis in colon cancer *via* platelet to lymphocyte ratio and platelet count. J Coll Physicians Surgeons–Pakistan JCPSP. (2020) 30(3):250–3. doi: 10.29271/jcpsp.2020.03.250 32169130

[23] Karpanen T Egeblad M Karkkainen MJ Kubo H Ylä-Herttuala S Jäättelä M. Vascular endothelial growth factor c promotes tumor lymphangiogenesis and intralymphatic tumor growth. Cancer Res (2001) 61(5):1786–90. 11280723

[24] Hirakawa S Brown LF Kodama S Paavonen K Alitalo K Detmar M. VEGF-c-induced lymphangiogenesis in sentinel lymph nodes promotes tumor metastasis to distant sites. Blood (2007) 109(3):1010–7. doi: 10.1182/blood-2006-05-021758 PMC1785149 17032920

[25] Harrell MI Iritani BM Ruddell A. Tumor-induced sentinel lymph node lymphangiogenesis and increased lymph flow precede melanoma metastasis. Am J pathology. (2007) 170(2):774–86. doi: 10.2353/ajpath.2007.060761 PMC1851877 17255343

[26] Zhou Z Wang P Sun R Li J Hu Z Xin H. Tumor-associated neutrophils and macrophages interaction contributes to intrahepatic cholangiocarcinoma progression by activating STAT3. J immunotherapy Cancer (2021) 9(3):e001946. doi: 10.1136/jitc-2020-001946 PMC7949476 33692217

[27] Zhang X Zhang W Yuan X Fu M Qian H Xu W. Neutrophils in cancer development and progression: Roles, mechanisms, and implications (Review). Int J Oncol (2016) 49(3):857–67. doi: 10.3892/ijo.2016.3616 27573431

[28] Buettner S Spolverato G Kimbrough CW Alexandrescu S Marques HP Lamelas J. The impact of neutrophil-to-lymphocyte ratio and platelet-to-lymphocyte ratio among patients with intrahepatic cholangiocarcinoma. Surgery (2018) 164(3):411–8. doi: 10.1016/j.surg.2018.05.002 29903509

[29] Schlesinger M. Role of platelets and platelet receptors in cancer metastasis. J Hematol Oncol (2018) 11(1):125. doi: 10.1186/s13045-018-0669-2 30305116 PMC6180572

[30] Reading JL Gálvez-Cancino F Swanton C Lladser A Peggs KS Quezada SA. The function and dysfunction of memory CD8(+) T cells in tumor immunity. Immunol Rev (2018) 283(1):194–212. doi: 10.1111/imr.12657 29664561

[31] Tan DW Fu Y Su Q Guan MJ Kong P Wang SQ. Prognostic significance of neutrophil to lymphocyte ratio in oncologic outcomes of cholangiocarcinoma: A meta-analysis. Sci Rep (2016) 6:33789. doi: 10.1038/srep33789 27694951 PMC5046177

[32] Goonetilleke KS Siriwardena AK. Systematic review of carbohydrate antigen (CA 19-9) as a biochemical marker in the diagnosis of pancreatic cancer. Eur J Surg Oncol J Eur Soc Surg Oncol Br Assoc Surg Oncol (2007) 33(3):266–70. doi: 10.1016/j.ejso.2006.10.004 17097848

