# Supplementary figures and images for: Association of systemic inflammation index with survival in patients with advanced perihilar cholangiocarcinoma treated with interventional therapy
Source: Front Oncol. 2022 Dec 22;12:1038759. doi: 10.3389/fonc.2022.1038759 (PMC9815453; doi:10.3389/fonc.2022.1038759)

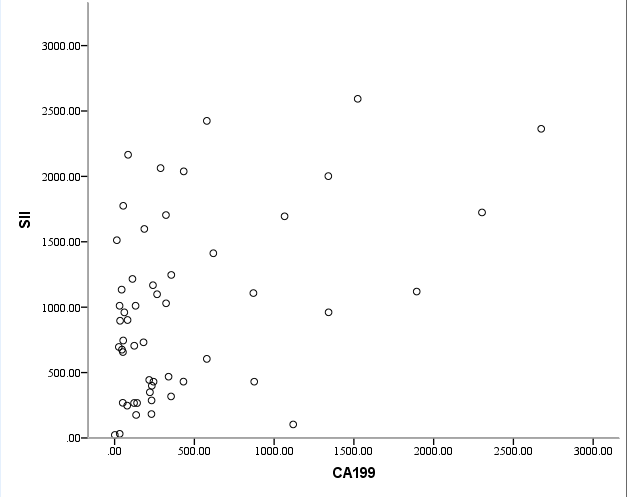

Supplement: Supplementary Figure 1 — Scatter plots between the SII and CA199 level. SII, systemic immune-inflammation index; CA199, carbohydrate antigen199. [file Image_1.png]
