# Supplementary material for: Association of systemic inflammation index with survival in patients with advanced perihilar cholangiocarcinoma treated with interventional therapy
Source: Front Oncol. 2022 Dec 22;12:1038759. doi: 10.3389/fonc.2022.1038759 (PMC9815453; doi:10.3389/fonc.2022.1038759)
Supplement: Supplementary file 2 [file Table_1.docx]

**Supplementary materials**

**Association of systemic inflammation index with survival in patients with advanced perihilar cholangiocarcinoma treated with interventional therapy**

*Jinyu Li ^1^† , Long Gao^1^†, Tianci Liu ^2^ †, Duiping Feng ^1 *^*

^1^ Department of Oncological and Vascular Intervention, First Hospital of Shanxi Medical University, Taiyuan, China

^2^ College of Medical Imaging, Shanxi Medical University, Taiyuan, China

^*^ Corresponding author.

†These authors contributed equally to this work.

Jinyu Li, email: [lijinyu92@126.com](mailto:lijinyu92@126.com);

Long Gao, email: gaolong@sxmu.edu.cn;

Tianci Liu, email: [1277125654@qq.com](mailto:1277125654@qq.com);

Duiping Feng, corresponding author, email: [fengdp@sxmu.edu.cn](mailto:fengdp@sxmu.edu.cn);

Table S1. Comparison of demographics and clinicopathological characteristics between Pre-PTCD and Pre-HAIC.

| Variables | Pre-PTCD | Pre-HAIC | P |
| --- | --- | --- | --- |
| Age (years)^a^ | 64.0 ± 1.2 | 64.0 ± 1.2 | - |
| Sex (Male) | 36 (54.5%) | 36 (54.5%) | - |
| ALT (U/L)^b^ | 110.5 (51.0, 134.0) | 53.5 (38.0, 110.3) | 0.004 |
| AST (U/L)^b^ | 120.0 (64.5, 159.3) | 60.5 (41.0, 107.3) | 0.001 |
| ALB (g/L)^b^ | 32.6 (29.1, 34.9) | 33.2 (28.8, 36.8) | 0.602 |
| TBIL (μmol/L)^b^ | 276.8 (185.5, 342.8) | 95.3 (33.0, 237.7) | <0.001 |
| CEA (μg/L)^b^ | 3.9 (1.9, 6.2) | 2.0 (0.8, 5.3) | 0.412 |
| CA199 (U/ml)^b^ | 305.1 (105.1, 1616.4) | 205.9 (46.5, 959.8) | 0.022 |
| CA125 (U/ml)^b^ | 33.5 (13.7, 80.6) | 53.5 (38.0, 110.3) | 0.469 |
| Tumor size (cm)^b^ | 3.2 (2.3, 4.9) | 3.2 (2.3, 4.9) | - |
| Lymph node metastasis (yes) | 37 (56.1%) | 37 (56.1%) | - |
| Vascular invasion (yes) | 13 (19.7%) | 13 (19.7%) | - |
| NLR^b^ | 4.2 (2.0, 7.1) | 3.3 (2.0, 4.8) | 0.100 |
| PLR^b^ | 190.4 (132.2, 270.0) | 161.3 (117.1, 221.6) | 0.023 |
| MLR^b^ | 0.5 (0.3, 0.8) | 0.5 (0.3, 0.7) | 0.793 |
| SII^b^ | 1049.2 (430.8, 1685.3) | 755.3 (376.8, 1080.7) | 0.002 |
| SIRI^b^ | 2.8 (1.0, 4.5) | 1.8 (0.9, 3.6) | 0.348 |

^a^ mean ± SD; ^b^ Median with inter-quartile range.

AST, aspartate aminotransferase; ALT, alanine aminotransferase; TBIL, total bilirubin; ALB, albumin; CEA, carcinoembryonic antigen; CA199, carbohydrate antigen199; CA125, carbohydrate antigen 125; NLR, neutrophil-lymphocyte ratio; PLR, platelet-lymphocyte ratio; MLR, monocyte-lymphocyte ratio; SII, systemic immune-inflammation index; SIRI, systemic inflammatory response index. Tumor size refers to the maximum diameter of the tumor; Lymph node metastasis and vascular invasion are evaluated by imaging.
